# NRO-stylization: A novel algorithm for regular octahedral stylization aesthetic modeling of three-dimensional surface mesh

**DOI:** 10.1371/journal.pone.0310242

**Published:** 2024-10-29

**Authors:** Fuxin Dong, Yingying Tang, Jialing Zhang, Kun Qian

**Affiliations:** 1 Faculty of Science, Kunming University of Science and Technology, Kunming, Yunnan, China; 2 Information School, Yunnan University of Finance and Economics, Kunming, Yunnan, China; Kwame Nkrumah University of Science and Technology, GHANA

## Abstract

Stylization of 3D shapes has been a prominent topic in computer graphics. Geometric stylization, as a category within 3D shape stylization, has garnered widespread attention due to its distinctive geometric aesthetics. This paper introduces a novel and simplified method for achieving regular octahedral stylization, capable of transforming the input shape into the regular octahedral style while preserving the content of the original shape. The proposed method achieves regular octahedral stylization by using face normals. Its implementation is straightforward because it incurs a cost only in terms of solving several linear systems. To demonstrate the effectiveness and robustness of the proposed method, extensive tests were conducted on models with varying levels of complexity and topological structures. The results highlight the exceptional capability of the proposed method to generate geometric shapes with an aesthetically pleasing regular octahedral style.

## Introduction

The stylization of 3D shapes has emerged as a prominent area of research within the field of computer graphics. Stylization is focused on defining a specific aesthetic or unique style through directed alterations based on geometric attributes. With the decreasing costs of 3D printing in recent years [1], there has been a renewed and growing interest among scholars in the modeling of non-realistic 3D shape stylization [2–7].

Several prominent geometric stylization methods have been developed for 3D shape stylization. In 2019, Liu and Jacobson proposed Cubic Stylization [2], which transformed input shapes into the style of a cube while retaining the original shape content. This method incorporates an *l*_1_ regularization term into the ARAP surface modeling approach [8] to align the rotated vertex normals with the coordinate axes. Subsequently, this technique has been expanded to incorporate arbitrary predefined target normals expressed via Gauss maps [3, 4]. In essence, these geometric stylization techniques are fundamentally aimed at achieving geometric stylization through the adoption of a local-global update strategy that minimizes the original ARAP energy [8] or the ARAP-Spoke-Rim [9] version along with additional terms introduced by each technique—see Background section for details.

**Fig 1:**
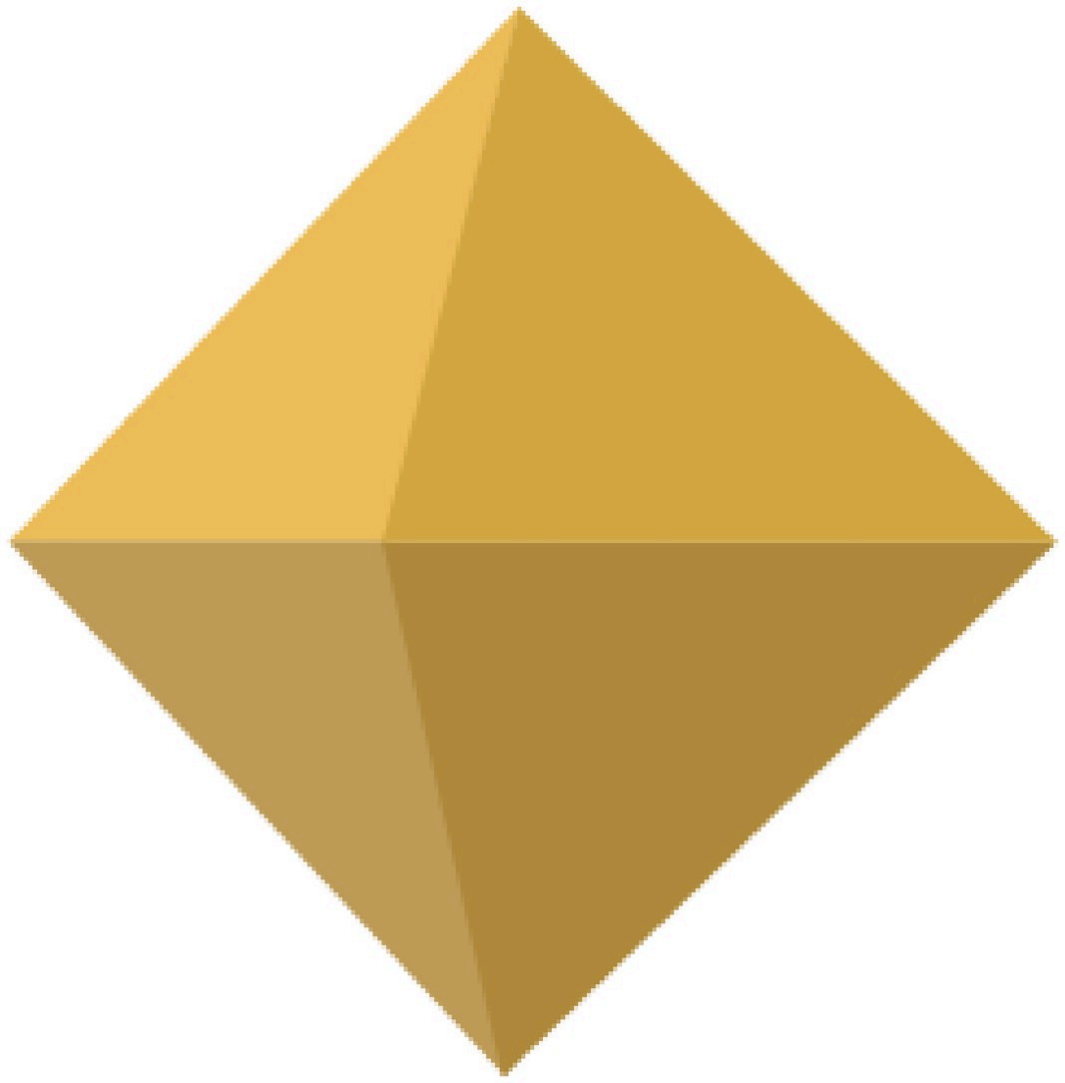
The regular octahedron.

The regular octahedron, known as a special type of convex polyhedron, consists of eight equilateral triangles, where each of its eight faces is an equilateral triangle and four faces at each vertex (Fig 1). Regular octahedra are commonly found in nature, with many natural crystals such as diamonds, alum, or fluorite exhibiting the characteristics of a regular octahedron. Additionally, the regular octahedron is prevalent in art and culture, with prominent examples such as the Egyptian pyramids, which can be conceived as the upper portion of a regular octahedron. Furthermore, the regular octahedron dice are among the more commonly encountered polyhedral dice.

**Fig 2:**
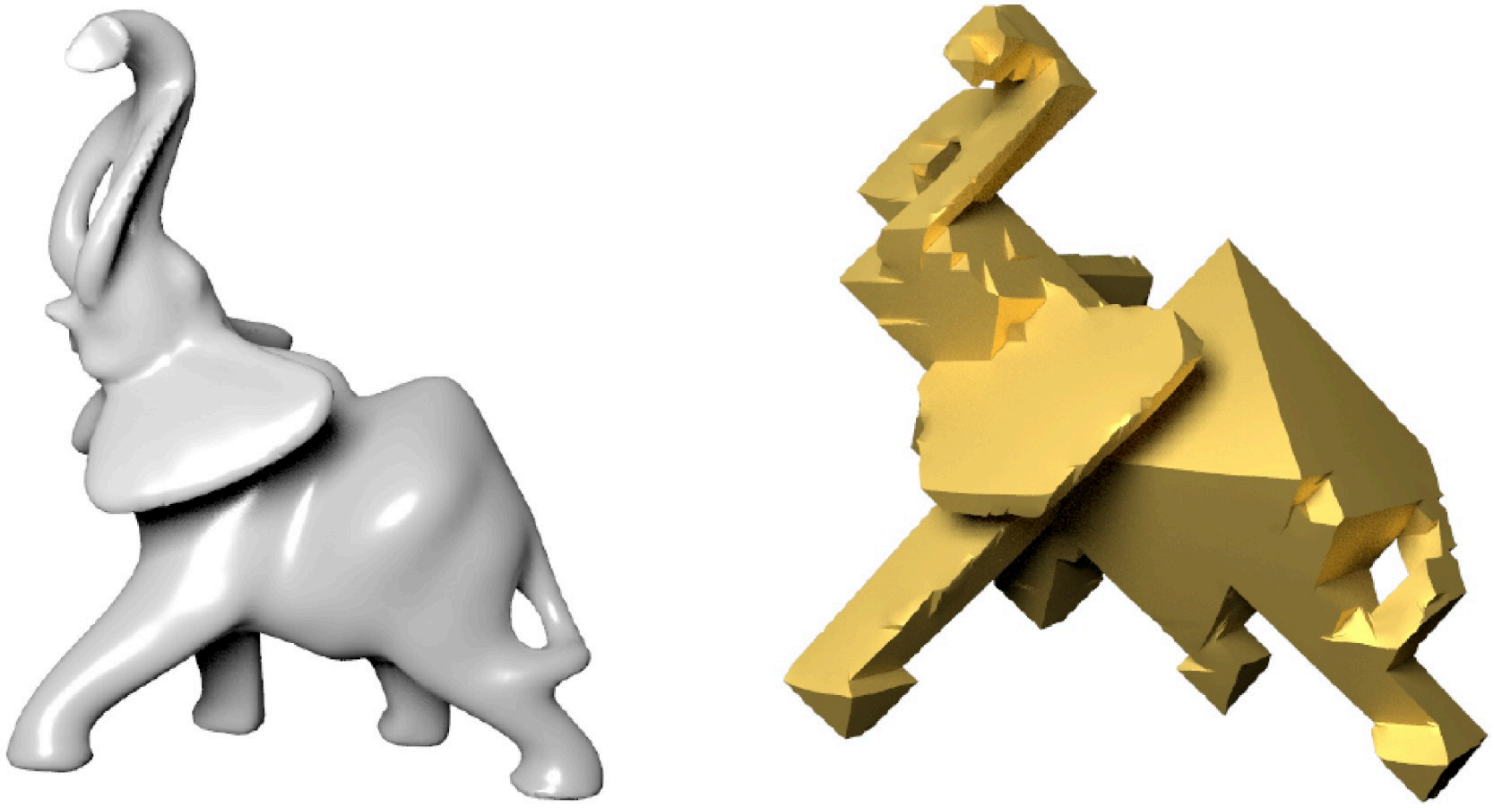
Modeling the input elephant model (left) in regular octahedral style (right) by using NRO-stylization.

In this paper, we introduce a novel approach for achieving regular octahedral stylization modeling(NRO-stylization). Similar to the Cubic Stylization proposed by Liu and Jacobson [2], NRO-stylization transforms the input shape into a regular octahedral style while preserving the original shape content. As shown in Fig 2, we contribute further to the enrichment of regular octahedral stylization by providing a modeling tool that takes 3D shapes as input and outputs meshes with the regular octahedral style.

Compared to the previous approach [3, 4] of utilizing a local-global update strategy [8] to minimize energy for achieving stylization, we simply need to solve several linear systems, making our method significantly simpler to implement than other approaches. We introduce a normal-driven Regular Octahedral Stylization involving the rotation of primitive face normals. It is worth noting that in order to capture the shape of the regular octahedral mesh, the rotation is determined by the original face normal and one of the eight face normals of the regular octahedron that is closest to the original face normal. NRO-stylization divides the modeling process of regular octahedral stylization into two simple steps: obtaining target normals and reconstructing the mesh with regular octahedral style. After computing the target face normals, all triangular faces are independently rotated from their original directions to align with the target normals. We employ a Poisson-based method [10–12] to reconstruct an effective manifold triangular mesh. The final problem is simplified into solving several linear systems to obtain the results. The proposed technology can handle various types of meshes, whether oriented or non-oriented, with or without boundaries. Compared to previous methods, the algorithm demonstrates equal efficiency and robustness, capable of transforming meshes with complex geometric shapes and arbitrary topology into their corresponding regular octahedral style.

The structure of the remaining sections of this paper is as follows. **Related Work** shows a brief overview of the progress related to aspects pertinent to the proposed method. **Background** presents a concise overview of the theoretical foundations and key ideas behind other geometric stylization techniques. **Methods** outlines the novel method for Regular Octahedral Stylization. We assess the feasibility of our method and conduct comparative analyses with previous approaches in **Results and Demonstrations**. Lastly, **Conclusions and Future Work** offers a summary of our findings and future directions.

## Related work

Our work shares the same motivation as that of Liu and Jacobson [2]. The main idea of NRO-stylization is to create a geometric representation with a regular octahedral appearance. That is, given an input shape, the objective is to preserve the shape content of the input model while transforming the input shape into a regular octahedral style. Therefore, the discussion will focus on methods for geometric processing.

### Geometric modeling-based stylization

Several notable geometric stylization methods. Cubic Stylization [2], which transforms input shapes into the style of a cube while retaining the original shape content. This method incorporates an *l*_1_ regularization term into the ARAP surface modeling approach [8] to align the rotated vertex normals with the coordinate axes. Subsequently, this technique has been expanded to incorporate arbitrary predefined target normals expressed via Gauss maps [3, 4]. The proposed method focuses on the field of 3D regular octahedral stylization, making contributions to enriching regular octahedral stylization.

### Normal-driven in shape deformation

Surface normals are a fundamental geometric quantity commonly used in geometry processing. Our main idea is to use surface normals to drive NRO-stylization. Many techniques, such as shape deformation for stylization [2–4, 6, 13], constructing shape abstractions [14], surface parameterization [12], and interactive mesh editing [15], are related to surface normals. One classic example is polycube deformation [16], where the goal is to optimize surface normals to be aligned with axes. Gregson et al., Huang et al., Fu et al. and Zhao et al. have used surface normals to create polycube shapes [10, 17–19]. Additionally, more examples can be found in the design of geometric filters, such as Guided filters [20], Shock filters [21], Bilateral normal filters [22], and Total Variation mesh denoising [23].

### Application of axis-alignment

Axis-alignment holds significant importance in various geometric processing tasks. Liu and Jacobson [2] proposed the addition of an *l*_1_ regularization term to promote alignment between rotated vertex normals and coordinate axes to achieve cubic stylization. Similarly, NRO-stylization encourages aligning the face normals with one of the eight face normals of the octahedron to capture its regular octahedral shape. Furthermore, this concept is one of the primary tools for constructing polycube mappings [10, 16–19, 24].

### Poisson system-based reconstruct

Poisson system-based deformation [15] is a widely used technique. Applying the pre-determining rotations of all initial triangle faces to rotate the triangular faces into new orientations, the Poisson system is used to blend triangles and the consistent mesh is reconstructed into the new shape. The interpolation of two corresponding meshes [25] or the utilization of constraint faces rotations [26] enables the attainment of rotations. In the context of mesh deformation [11], a local-global approach is employed for calculating the optimal rotations. In the construction of polycube [10], rotations are calculated based on the corresponding face normals of the polycube. In mesh parameterization [12], rotations are calculated based on the average face normals. NRO-stylization also relies on this approach, where we compute the rotation based on the original face normal and one of the eight face normals of the regular octahedron that is closest to the original face normal.

## Background

This section provides a brief overview of the theoretical foundations and main ideas behind several other geometric stylization techniques that are closest to the proposed approach.

### ARAP and ARAP-Spoke-Rim energy

Let *S* be the original surface and *S*′ be its deformed surface embedded in *R*^3^. We denote a 3-vector *x*_*p*_ as the position associated with the vertex *p* of *S*, and a 3-vector $x_{p}^{\prime }$ with the vertex *p*′ of *S*′. The half-edge from *p* to *q* is denoted as *he*_*pq*_, and the angle opposite to the half-edge *he*_*pq*_ is denoted as *θ*_*pq*_. The original ARAP energy [8] can be expressed as:

$$\begin{array}{c} \begin{array}{cc} E_{ARAP}\left(x^{\prime },R\right) & ≔\sum_{p}\sum_{q\in N(p)}[\text{cot}\left(\theta_{pq}\right)+\text{cot}\left(\theta_{qp}\right)]\\ & \left|\right|\left(x_{p}^{\prime }-x_{q}^{\prime }\right)-R\left(p\right)\left(x_{p}-x_{q}\right){\left|\right|}^{2}\\ & =\sum_{he_{pq}}[\text{cot}\left(\theta_{pq}\right)+\text{cot}\left(\theta_{qp}\right)]\\ & \left|\right|\left(x_{p}^{\prime }-x_{q}^{\prime }\right)-R\left(p\right)\left(x_{p}-x_{q}\right){\left|\right|}^{2} \end{array} \end{array}$$ (1)

Here *N*(*p*) is defined as the set of vertices adjacent to vertex *p*. *R*(*p*) denotes a 3 × 3 rotation matrix defined on *p*.

The ARAP-Spoke-Rim energy [9] is as follows:

$$\begin{array}{c} \begin{array}{cc} E_{ARAP-SR}\left(x^{\prime },R\right) & ≔\sum_{p}\sum_{\left(r,s)\in E(p\right)}[\text{cot}\left(\theta_{rs}\right)+\text{cot}\left(\theta_{sr}\right)]\\ & \left|\right|\left(x_{r}^{\prime }-x_{s}^{\prime }\right)-R\left(p\right)\left(x_{r}-x_{s}\right){\left|\right|}^{2} \end{array} \end{array}$$ (2)

Here *E*(*p*) is defined as the set of all of the edges in the triangles adjacent to *p*.

### Other geometric stylization

Liu and Jacobson [2] proposed an idea of cubic stylization, in which they minimized the energy composed of *l*_1_ regularization on the surface normals and the ARAP term. This method transforms the input shape into a cubic style while preserving the content of the original shape. The work [2] minimizes the energy formula as follows:

$$\begin{array}{c} \begin{array}{c} E=E_{ARAP}\left(x^{\prime },R\right)+\lambda \sum_{p}a_{p}\left|\right|R_{p}{\hat{n}}_{p}{\left|\right|}_{1} \end{array} \end{array}$$ (3)

Where ${\hat{n}}_{p}$ is the original vertex normals, *a*_*p*_ is the barycentric area associated to vertex *p*. *l*_1_ regularization promotes the alignment between the rotated vertex normals and the coordinate axes. The parameter *λ* achieves a delicate balance between normal constraint and preservation of the original geometry.

Recently, other geometric stylization techniques [3, 4] have extended to arbitrary pre-defined target normals represented through Gauss maps. Similar to Cubic Stylization [2], Gauss Stylization [4] minimizes the energy composed of the original ARAP energy and the term penalizes the deviation between the surface normals and the preferred normal directions. Spherical shape analogies (SSA) [3] is to minimize the energy as follows:

$$\begin{array}{c} \begin{array}{c} E=E_{ARAP-SR}\left(x^{\prime },R\right)+\lambda a_{P}\|R_{P}{\hat{n}}_{P}-t_{P}{\|}_{2}^{2} \end{array} \end{array}$$ (4)

Where *R*(*p*) is a 3 × 3 rotation matrix defined on *p*, and ${\hat{n}}_{p}$ is the *p*th unit vertex normal of the input mesh computed via area-weighted average of face normals. The target normals *t*_*p*_ for each vertex *p* encode the geometric style. The parameter λ also delicately balances between normal constraints and preserving the original geometric shape.

Furthermore, SSA [3] has also been extended to incorporate different regularizations, such as *Face-only* ARAP. It allows two adjacent triangles to bend freely, resulting in sharp creases.

$$\begin{array}{c} \begin{array}{cc} E_{FARAP}\left(x^{\prime },R\right) & ≔\sum_{f}\sum_{\left(i,j\right)\in v_{f}}[\text{cot}\left(\theta_{ij}\right)+\text{cot}\left(\theta_{ji}\right)]\\ & \left|\right|\left(x_{i}^{\prime }-x_{j}^{\prime }\right)-R\left(f\right)\left(x_{i}-x_{j}\right){\left|\right|}^{2} \end{array} \end{array}$$ (5)

Where *f* is the initial triangle face, and *v*_*f*_, *R*(*f*) for the three edge vectors and the rotation matrix of the face *f* respectively.

Different from NRO-stylization of defining rotation matrices on faces, the methods of Cubic Stylization [2], Gauss Stylization [4], and SSA [3] (excluding its Face-only ARAP version) define rotation matrices based on points. In their approach, the rotation matrices are unknown variables that change at each iteration, whereas in our method, rotation matrices are predetermined and remain constant throughout each iteration.

**Fig 3:**
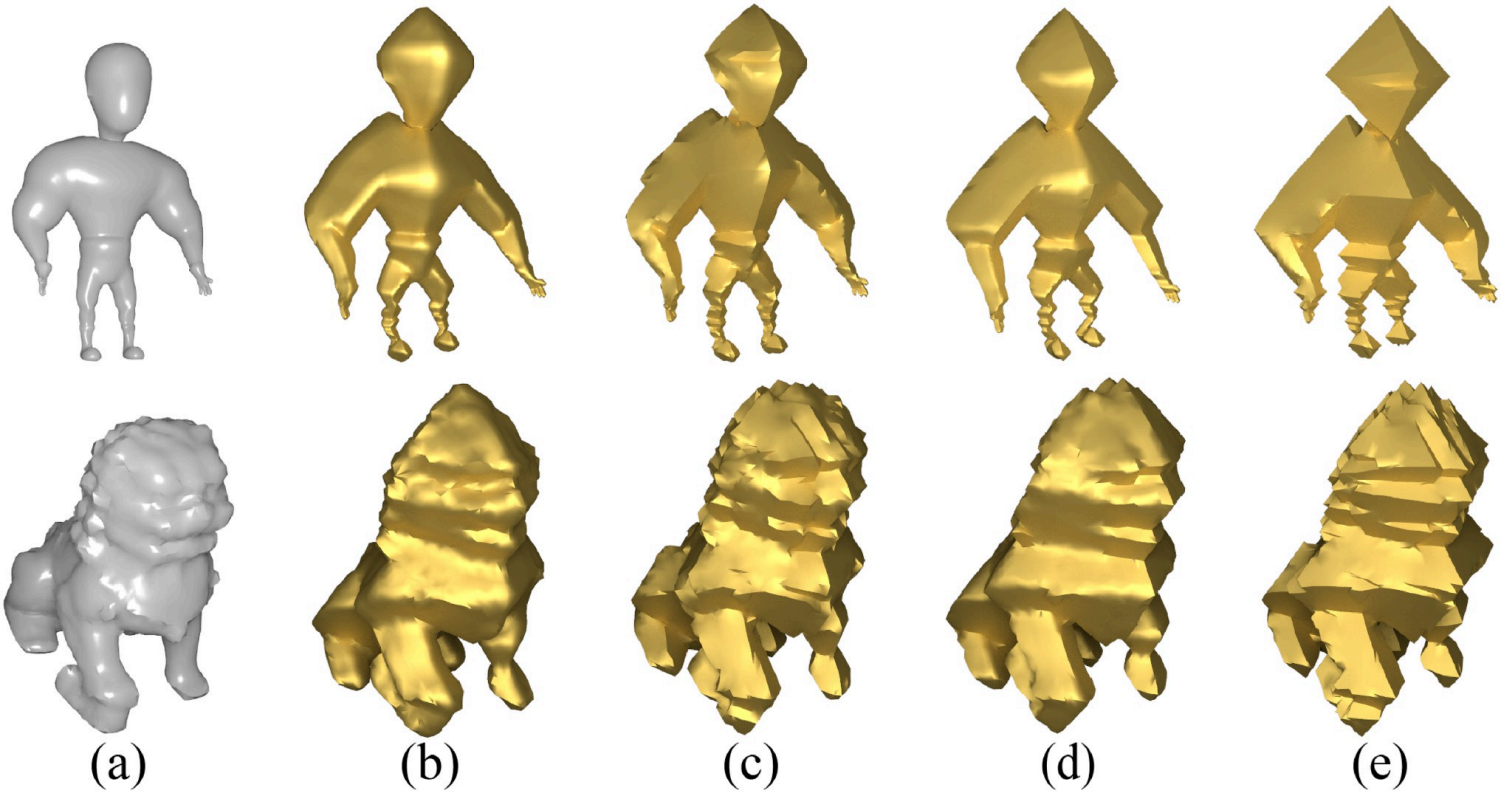
Visual comparison of regular octahedral stylization under different methods. Images (a) are original meshes; (b) ARAP-Spoke-Rim version of SSA [3]; (c) *Face-only* ARAP version of SSA [3]; (d) Gauss stylization [4]; (e) NRO-stylization.

Furthermore, fundamentally, these geometric stylization techniques fundamentally aimed at achieving stylization through the adoption of a local-global update strategy [8] that minimizes the original ARAP energy or the ARAP-Spoke-Rim version along with additional terms introduced by each technique. Comparing to other methods, as illustrated in Fig 3, we have employed a more simplified approach to achieve the regular octahedral stylization of modeling similar to these methods.

## Methods

The proposed approach takes as input a model of a general shape and outputs a regular octahedral style geometric object. The main idea is to use face normals to drive NRO-stylization. The algorithm consists of two simple steps:

- Obtaining target normals from the current face normals.
- Reconstructing the surface by the constraints of the current face normals.

### Target normals

Given the goal of our study to achieve regular octahedral stylization, we advocate aligning the normals of the triangular faces of the input mesh with those of the faces of a regular octahedron to capture its regular octahedral shape. Thus, it is necessary to first compute the normal vectors for each face of the regular octahedron.

**Fig 4:**
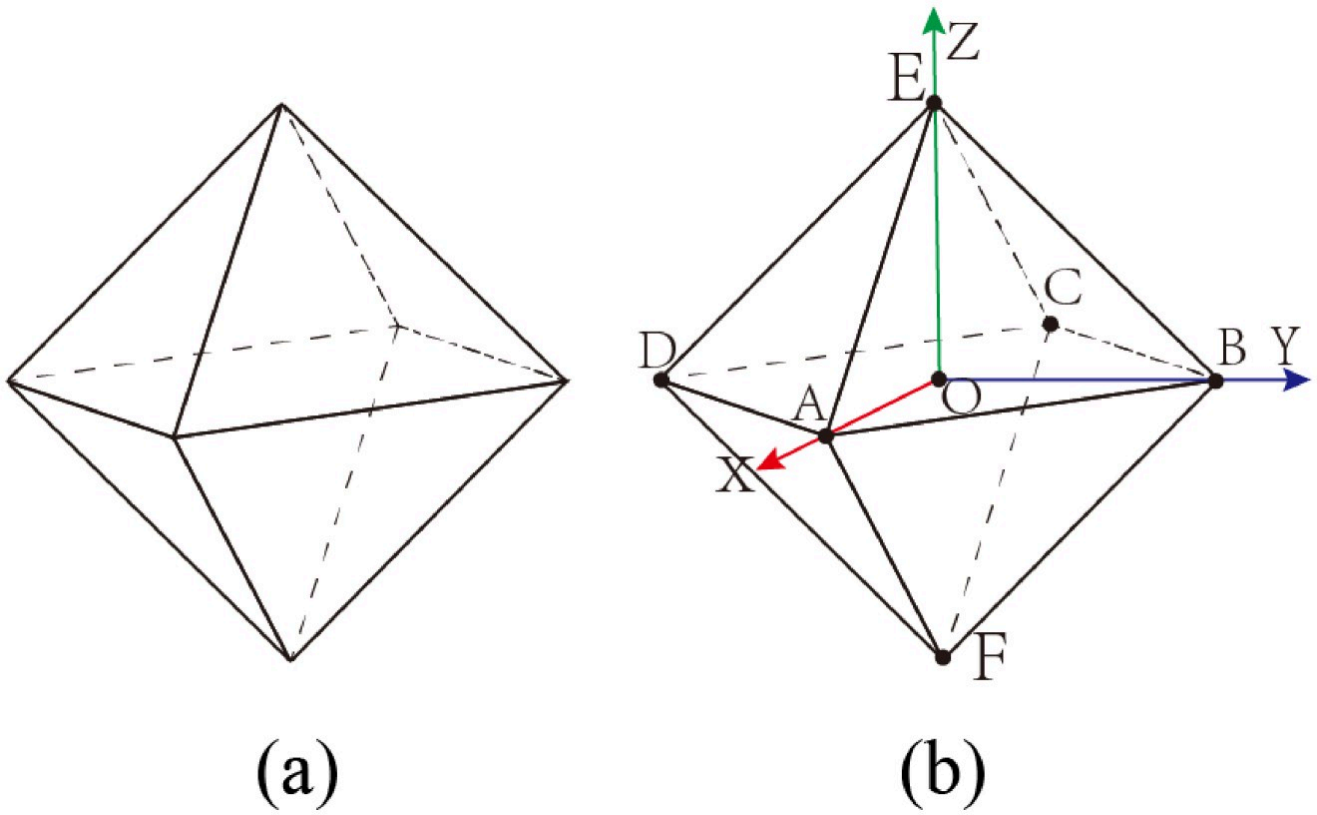
(a) Spatial schematic of a regular octahedron; (b) Establishment of a Cartesian coordinate system based on (a).

Leveraging the topology properties of the regular octahedron, as depicted in Fig 4, we establish a Cartesian coordinate system with the center of the regular octahedron as the origin *O* (also the intersection point of vectors *CA* and *DB*). Since the quadrilateral *ABCD* is a square, its diagonals are perpendicular to each other. Therefore, we define the direction of vector *OA* as the positive X-axis (red) and the direction of vector *OB* as the positive Y-axis (blue). It is also evident that the vector *OE* is perpendicular to the quadrilateral *ABCD*, thus the direction of vector *OE* can be defined as the positive Z-axis (green). Assuming an edge length of $\sqrt{2}$ for the regular octahedron, the coordinates of its six vertices are delineated as follows: *A*(1, 0, 0), *B*(0, 1, 0), *C*(−1, 0, 0), *D*(0, −1, 0), *E*(0, 0, 1), *F*(0, 0, −1).

**Fig 5:**
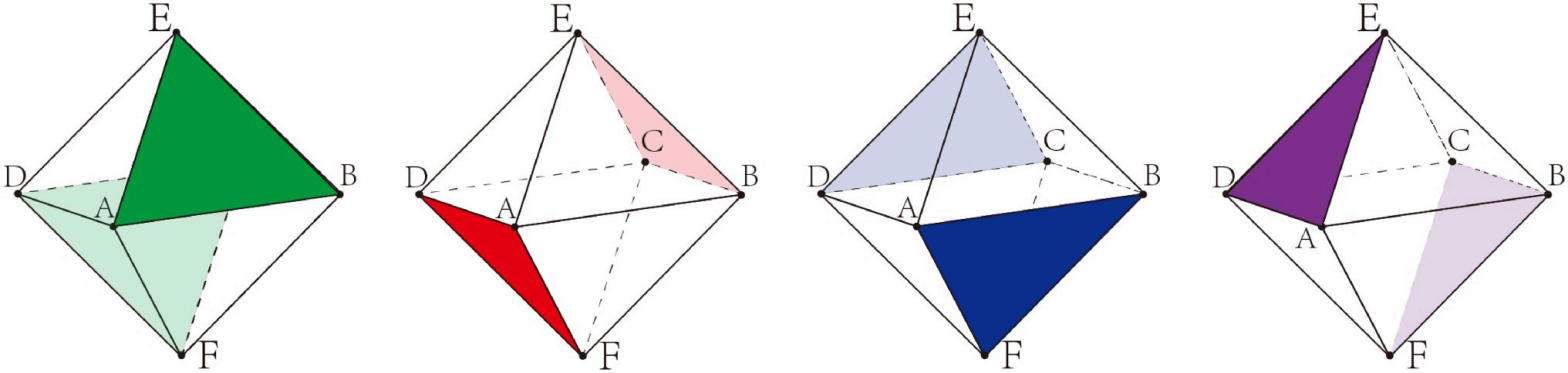
Four pairs of parallel planes in the regular octahedron.

As we obtain the coordinates of the six vertices of the regular octahedron, we can compute the normal vectors of its eight faces. However, in practical computations, it is unnecessary to compute the normal vectors for all eight faces, as depicted in Fig 5, where the eight faces of the regular octahedron are pairwise parallel. In 3D models, there is a distinction between inner and outer surfaces. Strictly speaking, the dihedral angle between two parallel faces is 180° in Fig 5, resulting in the normals of the paired parallel faces having opposite signs.

Therefore, we only need to calculate the normals of the four faces of the regular octahedron located above the *xy-plane*; the normals of the remaining faces can be obtained by simply adding a negative sign to the normals of their parallel face. After normalizing the normals of the eight faces of the regular octahedron, we obtain the following set *N* of normals:

$$\begin{array}{c} N\{\begin{array}{cc} & n_{faceABE}=\left(\frac{\sqrt{3}}{3},\frac{\sqrt{3}}{3},\frac{\sqrt{3}}{3}\right)\\ & n_{faceBCE}=\left(-\frac{\sqrt{3}}{3},\frac{\sqrt{3}}{3},\frac{\sqrt{3}}{3}\right)\\ & n_{faceCDE}=\left(\frac{\sqrt{3}}{3},\frac{\sqrt{3}}{3},-\frac{\sqrt{3}}{3}\right)\\ & n_{faceADE}=\left(-\frac{\sqrt{3}}{3},\frac{\sqrt{3}}{3},-\frac{\sqrt{3}}{3}\right)\\ & n_{faceCDF}=\left(-\frac{\sqrt{3}}{3},-\frac{\sqrt{3}}{3},-\frac{\sqrt{3}}{3}\right)\\ & n_{faceADF}=\left(\frac{\sqrt{3}}{3},-\frac{\sqrt{3}}{3},-\frac{\sqrt{3}}{3}\right)\\ & n_{faceABF}=\left(-\frac{\sqrt{3}}{3},-\frac{\sqrt{3}}{3},\frac{\sqrt{3}}{3}\right)\\ & n_{faceBCF}=\left(\frac{\sqrt{3}}{3},-\frac{\sqrt{3}}{3},\frac{\sqrt{3}}{3}\right) \end{array} \end{array}$$ (6)

After obtaining the set *N* of normal vectors for the regular octahedron, we identify from *N* the normal closest to the normal of the original triangular face and designate it as the target normals for alignment. Thus, the formula for the target normal of the original triangular face *i* is as follows:

$$\begin{array}{c} n_{i}\left(t+1\right)=N_{n_{i}\left(t\right)} \end{array}$$ (7)

where *N* denotes the set from Eq (6), and the *n*_*i*_(*t*) denotes the normal of face *i* at time *t*, *N*_*n*_*i*_(*t*)_ representing the normal in *N* that is closest to *n*_*i*_(*t*). Then the new normal at time *t* + 1 is *n*_*i*_(*t* + 1).

### Reconstruct surface

#### Poisson system-based reconstruct

After computing the new face normals via the first step above, we rotate all triangle faces from their old orientations to the current ones independently. However, the resulting triangle set did not form a valid mesh. To reconstruct an effective manifold triangular mesh, we employed a Poisson system-based approach [10–12]. This step can be regarded as solving a system of unknown positions based on known normal variables.

Let *S* be the original surface and *S*′ be its deformed surface embedded in *R*^3^. We denote a 3-vector *x*_*p*_ be the position associated with vertex *p* of *S*, and a 3-vector $x_{p}^{\prime }$ with vertex *p*′ of *S*′. On each triangle mesh *T*, we define a rotation matrix *R*(*T*). The half-edge from *p* to *q* is denoted as *he*_*pq*_, and the angle opposite to the half-edge *he*_*pq*_ is denoted as *θ*_*pq*_, and *R*(*T*_*pq*_) representing the rotation matrix associated with the triangle face containing half edge *he*_*pq*_. Zhao et al. [11] define the stretching energy as follows:

$$\begin{array}{c} E_{s}\left(x^{\prime },R\right)=\sum_{he_{pq}}\text{cot}\left(\theta_{pq}\right)\left|\right|\left(x_{p}^{\prime }-x_{q}^{\prime }\right)-R\left(T_{pq}\right)\left(x_{p}-x_{q}\right){\left|\right|}^{2} \end{array}$$ (8)

While this energy is similar to the original ARAP energy, the ARAP-Spoke-Rim version and the *Face-only* ARAP, there are differences in detail.

By computing the gradient of the stretching energy in Eq (8) and setting it to zero [10], after fixing a vertex, we can obtain the unknown position variable *x*′ by solving a single linear system as shown below [9, 11, 27]:

$$\begin{array}{c} \begin{array}{cc} & \sum_{q\in N(p)}[\text{cot}\left(\theta_{pq}\right)+\text{cot}\left(\theta_{qp}\right)]\left(x_{p}^{\prime }-x_{q}^{\prime }\right)\\ & =\sum_{q\in N(p)}[\text{cot}\left(\theta_{pq}\right)R\left(T_{pq}\right)+\text{cot}\left(\theta_{qp}\right)R\left(T_{qp}\right)]\left(x_{p}-x_{q}\right) \end{array} \end{array}$$ (9)

By defining the 3-vector at vertex *p* as follows:

$$\begin{array}{c} b_{p}=\sum_{q\in N(p)}[\text{cot}\left(\theta_{pq}\right)R\left(T_{pq}\right)+\text{cot}\left(\theta_{qp}\right)R\left(T_{qp}\right)]\left(x_{p}-x_{q}\right) \end{array}$$ (10)

We can rewrite the above system into the following matrix format:

$$\begin{array}{c} Lx^{\prime }=b \end{array}$$ (11)

where *L* is the *n* × *n* Laplacian matrix, and *x*′ and *b* is a *n* × 3 matrix.

By using of the current face normal and its corresponding target face normal, we can compute the rotation matrix *R*(*T*) between them through Rodrigues’ formula. Next, we solve Eq (11) by fixing the position of one vertex.

#### Iteration

**Fig 6:**
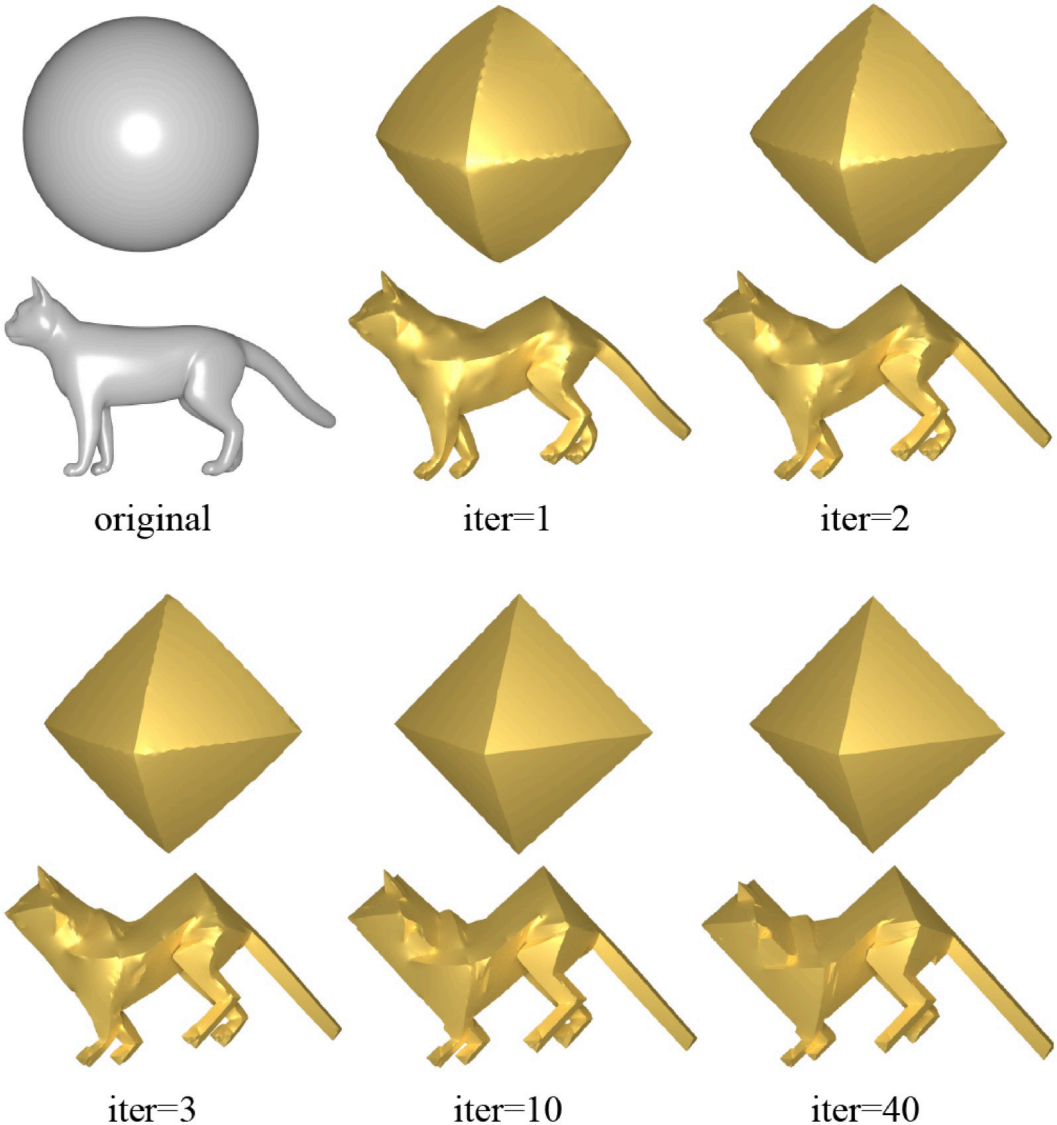
Controlling the degree of regular octahedral stylization of the original model through varying iterations.

As outlined in **Poisson system-based reconstruct**, NRO-stylization is based on Poisson system reconstruction deformation to meet the specified target face normals. Since the Poisson system can only approximate the input normal requirements, the system in Eq (11) will not generate an exact regular octahedral style as depicted in Fig 6. However, after applying the preceding steps, we obtain a new mesh that exhibits a geometric style closer to that of a regular octahedron compared to the original mesh. Thus, we consider the role of iteration by using an iterative Poisson system. After multiple iterations, the system converges and outputs the shape of the corresponding regular octahedral style.

Therefore, in algorithm, we control the degree of the regular octahedral style of the input model through varying iteration counts. Because a higher number of iterations tends to result in a stronger regular octahedral style, often leading to loss of detail in the input mesh. In the method for a regular octahedral style, our goal is to retain the details of the input mesh while achieving regularization in the output mesh. Therefore, we use the iteration count as a parameter to control the balance between the two. In each step *i*, we recompute the rotation matrix *R*_*i*_(*T*) by utilizing the face normal of the current model and the target normal provided by the regular octahedral topology. By using the new *R*_*i*_(*T*), we update the *L*_*i*_ and *b*_*i*_. The iterative process can be represented as:

$$\begin{array}{c} L_{i}x_{i}^{\prime }=b_{i} \end{array}$$ (12)

## Results and demonstrations

In this part, we initially evaluate the feasibility of our method in various meshes with complex topology, with and without boundaries. Additionally, a comparison is made between the proposed approach and previous methods. The proposed algorithms are implemented using generic C sharp under Windows Visual Studio. The performing computer is equipped with a Core i7–10750H CPU and 16GB of memory.

### Evaluation

The proposed method was tested on various meshes with complex topology and boundaries. The experimental results demonstrate the robustness of the algorithm, as it effectively handles all types of shapes.

**Fig 7:**
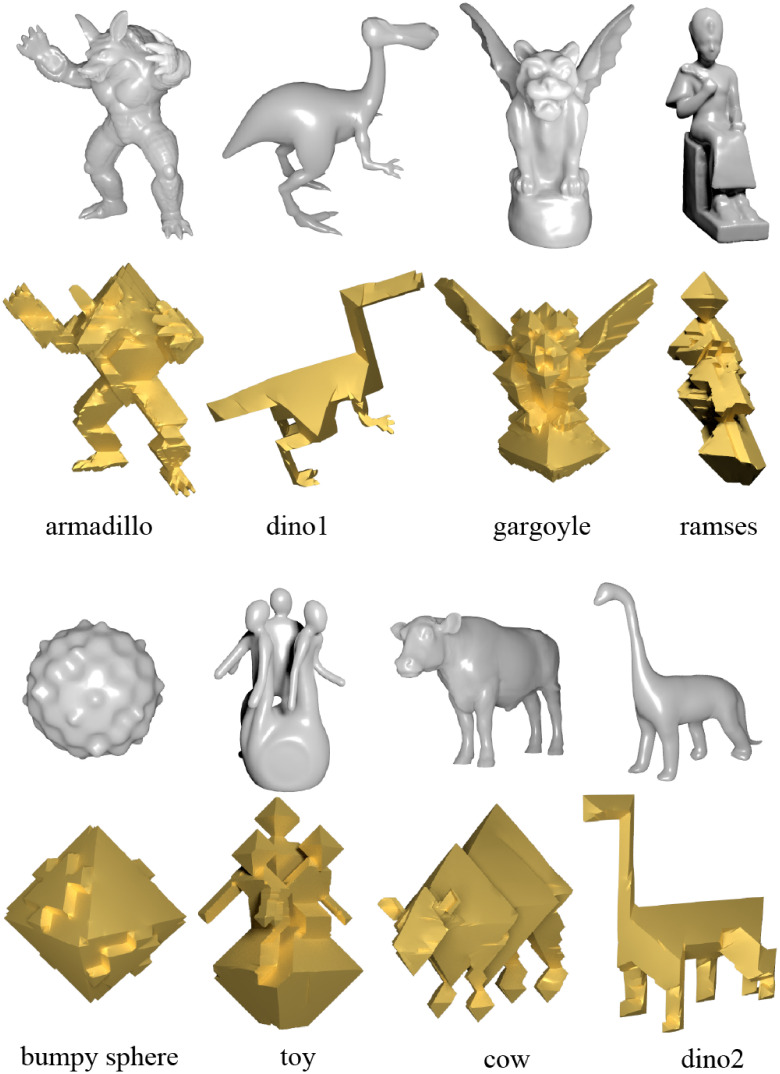
Eight models and their regular octahedral style shapes.

In Fig 7, we present several models along with their shapes in the regular octahedral style. The results indicate that the proposed algorithm can successfully achieve the regular octahedral style, regardless of the complexity of the model shapes.

**Fig 8:**
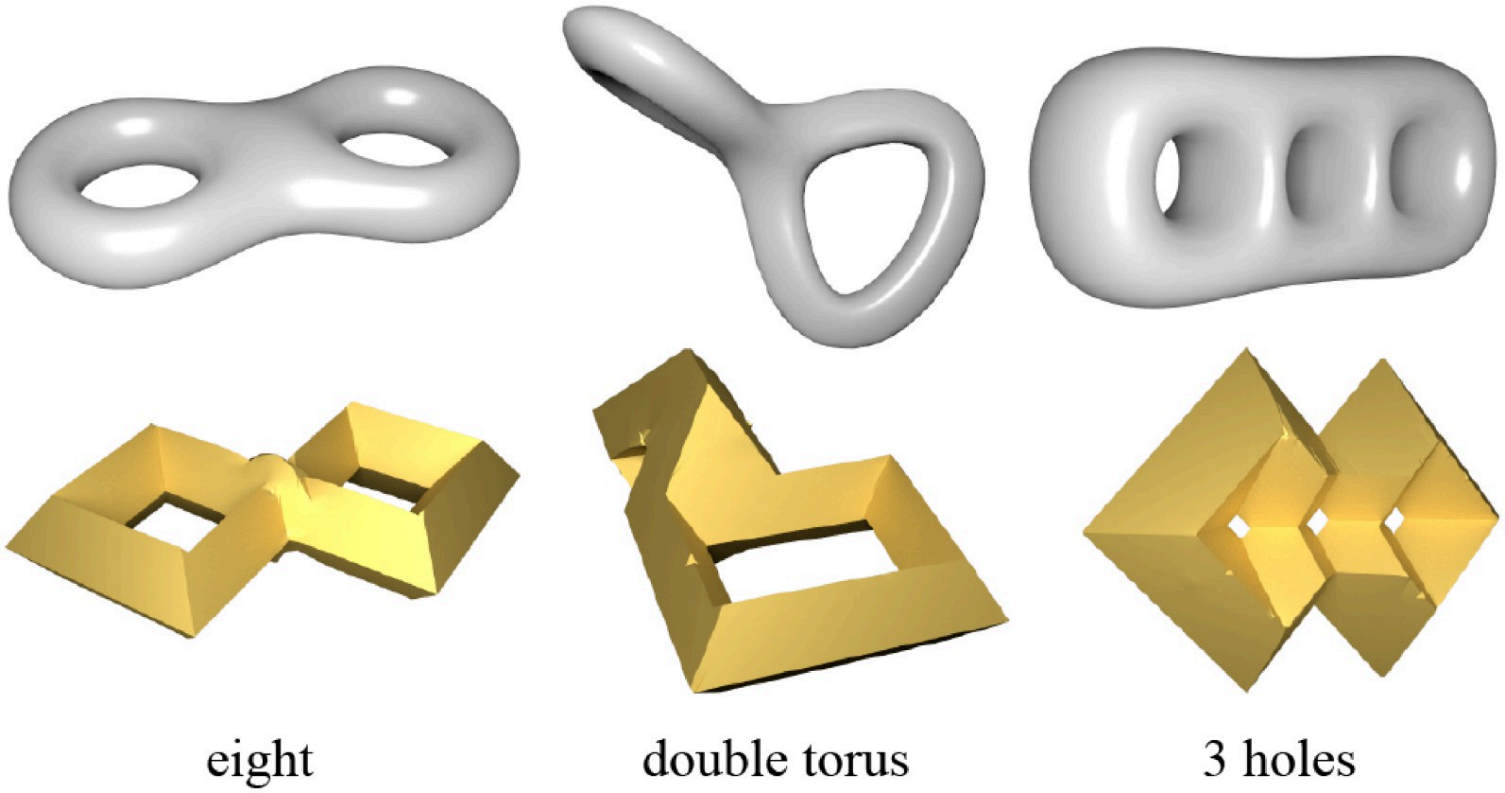
High genus models and their regular octahedral style shapes.

**Fig 9:**
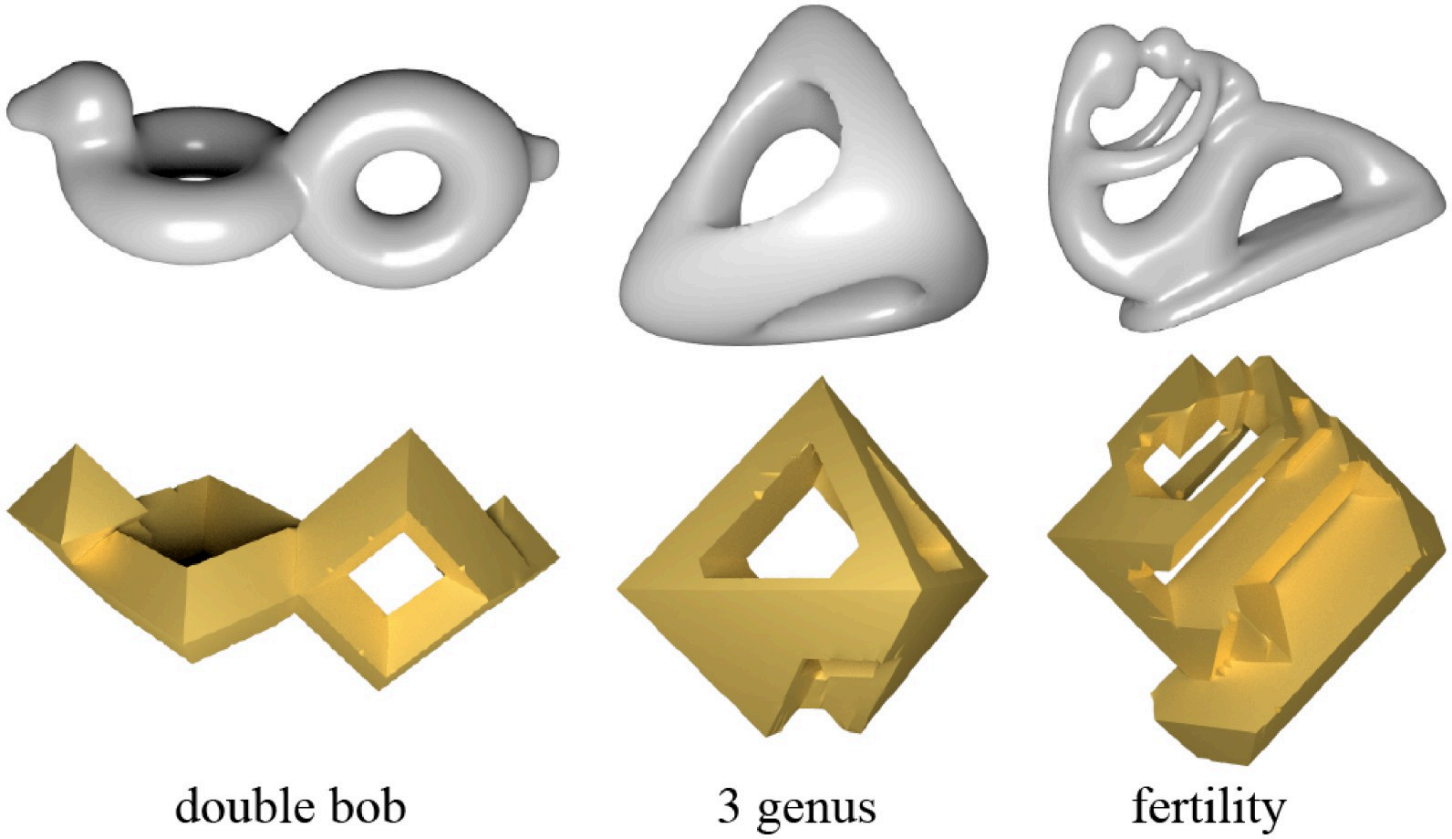
High genus models and their regular octahedral style shapes.

The proposed technique demonstrates excellent applicability to high-genus meshes. As shown in Figs 8 and 9, the technique can be directly applied to models with high genus, demonstrating the robustness of the algorithm to different mesh topologies.

**Fig 10:**
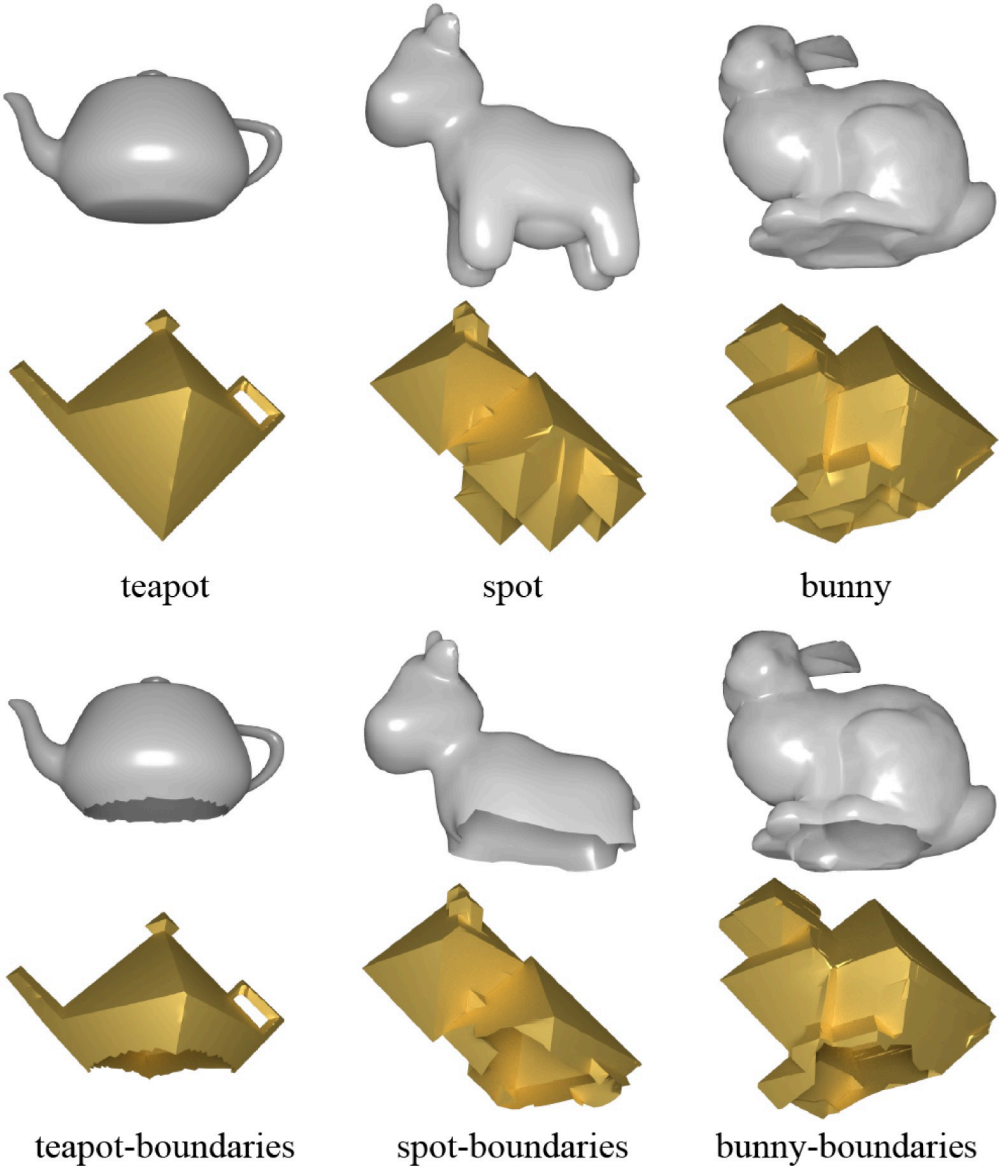
Models with and without boundaries, and their regular octahedral style shapes. The teapot models with and without boundaries underwent 20 iterations, the spot models with and without boundaries underwent 50 iterations, and the bunny models with and without boundaries underwent 100 iterations.

The approach is insensitive to the presence of boundaries. To assess its sensitivity to boundaries, we removed some faces from models without boundaries, converting them into models with boundaries. As illustrated in Fig 10, the approach remains unaffected by boundaries, thereby demonstrating its robustness in handling models with boundaries.

**Fig 11:**
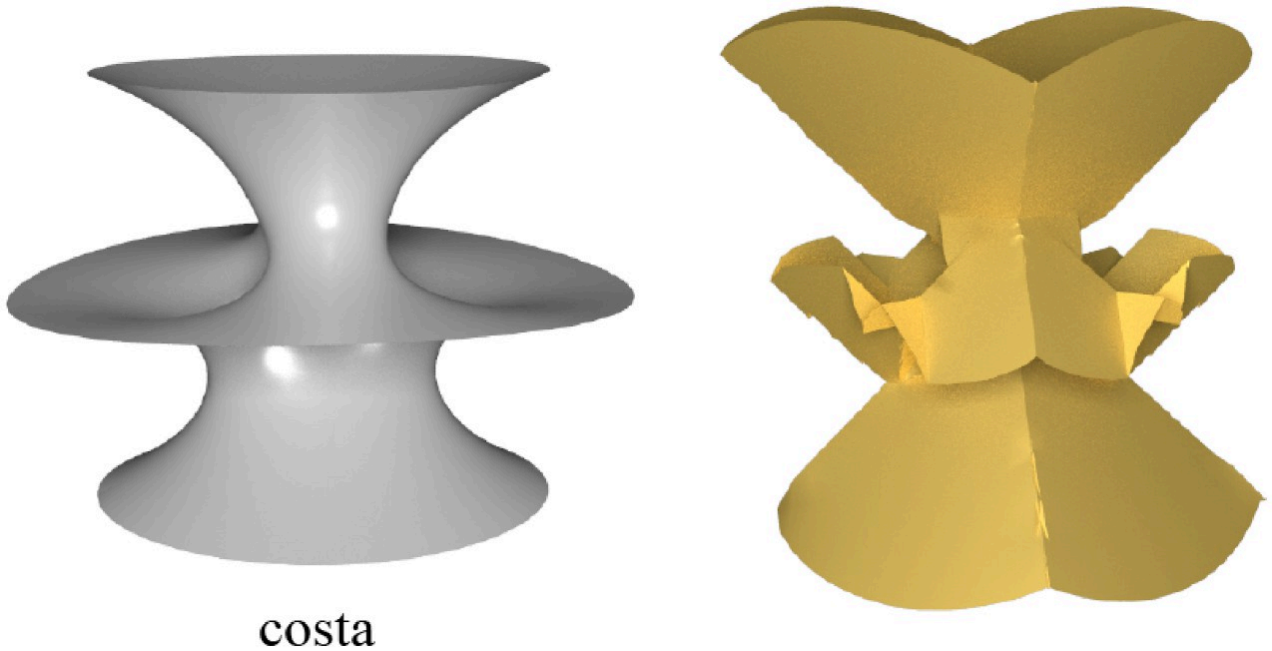
Costa, a non-orientable surface, and its regular octahedral stylization meshes.

We demonstrate the successful application of the algorithm to non-orientable meshes. In Fig 11, the approach transforms the well-known “costa” surface mesh, known for its non-orientable nature, into a mesh with a regular octahedral style.

### Comparison

We compared NRO-stylization with other techniques capable of modeling regular octahedral stylization, primarily contrasting the ARAP-Spoke-Rim version and the *Face-only* ARAP version of spherical shape analogies (SSA) proposed by Liu and Jacobson [3], Gauss Stylization proposed by Kohlbrenner et al. [4], these three being state-of-the-art methods.

**Fig 12:**
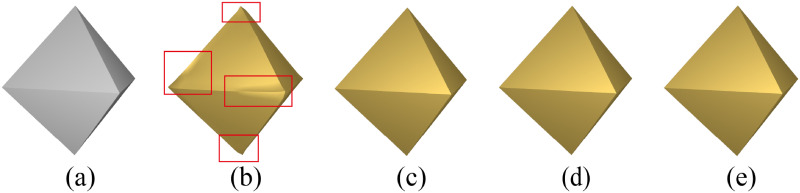
Regular octahedral stylization of a regular octahedron under different methods. Image (a) is the original regular octahedron model; (b) ARAP-Spoke-Rim version of SSA [3]; (c) *Face-only* ARAP version of SSA [3]; (d) Gauss stylization [4]; (e) NRO-stylization.

Different choices of normals can result in some discrepancies. In the “regular octahedral stylization of a regular octahedron” illustrated in Fig 12, the ARAP-Spoke-Rim version of SSA [3] utilizes a penalty function based on vertex normals, resulting in its geometric alteration of the perfectly regular octahedron shape, with unexpected transitions occurring at sharp edges. In contrast, Gauss Stylization [4], the *Face-only* ARAP version of SSA [3], and NRO-stylization are all based on face normals, preserving shapes that already adhere to the desired style.

**Fig 13:**
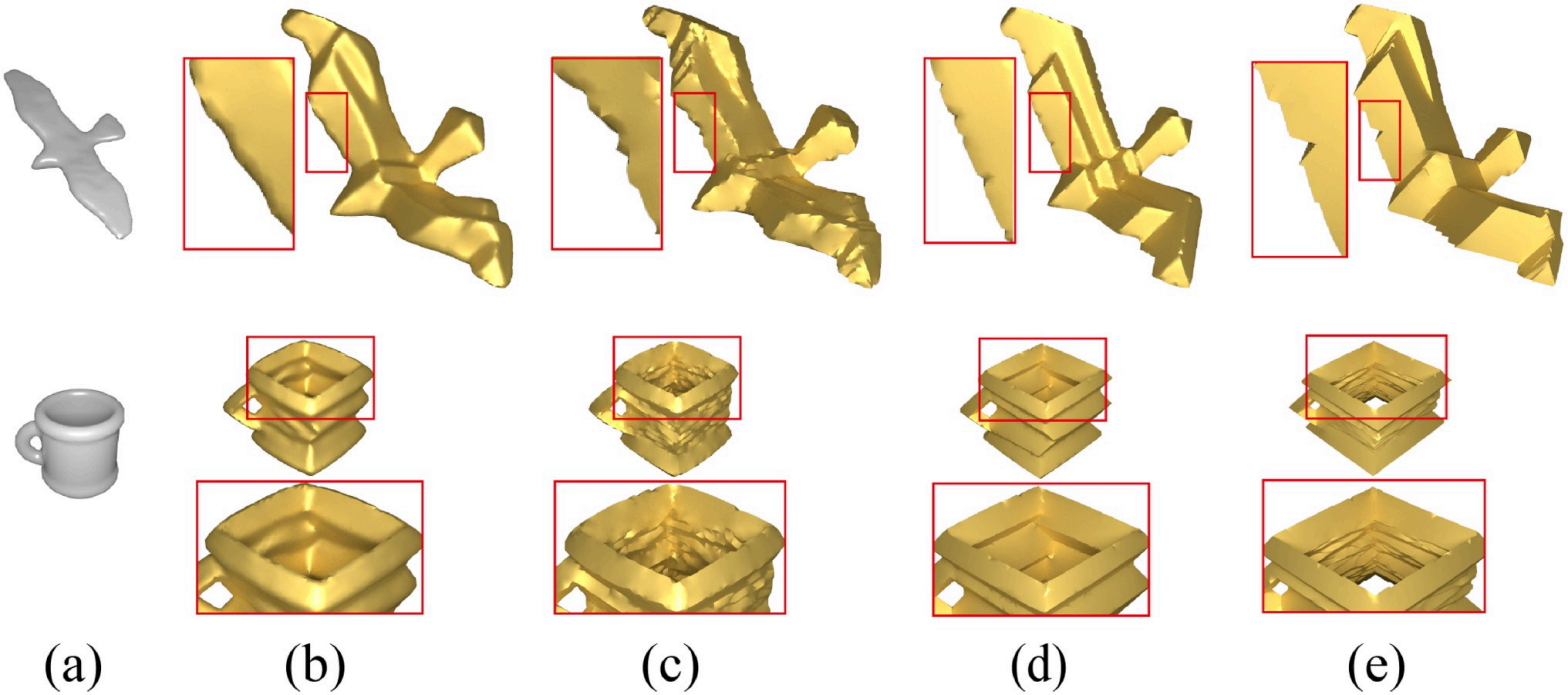
Comparison of edges handling for some models by different methods. Images (a) are models; (b) ARAP-Spoke-Rim version of SSA [3]; (c) *Face-only* ARAP version of SSA [3]; (d) Gauss stylization [4]; (e) NRO-stylization.

In dealing with the edges of some meshes, as illustrated in Fig 13, the face-based methods by Gauss Stylization [4], the *Face-only* ARAP version of SSA [3], and NRO-stylization tends to result in more prominent edges. The vertex normals in the ARAP-Spoke-Rim version of SSA [3] tend to smooth the result.

**Fig 14:**
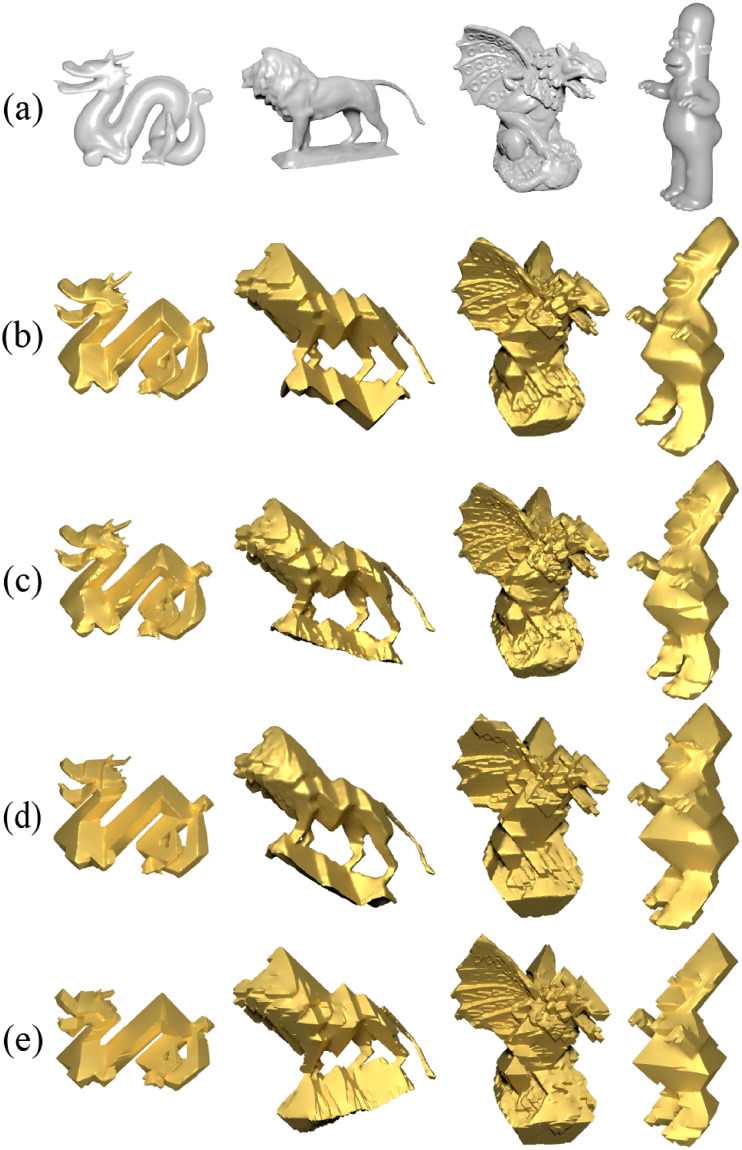
Visual comparison of octahedral stylization across various methods for some models. Images (a) are original models; (b) ARAP-Spoke-Rim version of SSA [3]; (c) *Face-only* ARAP version of SSA [3]; (d) Gauss stylization [4]; (e) NRO-stylization.

We extensively compared NRO-stylization with other approaches and presented several results in Fig 14. We found that both NRO-stylization and the *Face-only* ARAP version of SSA [3] remove the bending term, allowing adjacent triangles to bend freely, thus permitting a non-smooth deformation. A clear example is their treatment of the base of the “lion” model in Fig 14.

Overall, while several methods exhibit slight differences in generating the regular octahedral stylization, the overall styles are generally similar. In terms of algorithm implementation, NRO-stylization differs from others in that it only requires solving several linear systems without adopting a local-global update strategy to minimize energy. Therefore, compared to other methods, The proposed method can be simply implemented.

## Conclusions and future work

This paper presents a robust and straightforward method for driving regular octahedral stylization by using face normals. The primary distinction of the proposed method from other methods is that it does not require the implementation of a local-global update strategy to minimize energy. Instead, the proposed method only requires solving several linear systems, making it remarkably simple to implement in comparison with others. Furthermore, extensive tests have been conducted on meshes with various topological structures and different levels of complexity to demonstrate its effectiveness in handling all types of meshes. The planar quadrilateral (PQ) mesh with lower construction costs [28] has emerged as an appealing approach for achieving freeform modeling of architectural glass structures. In recent years, several techniques have been proposed for constructing PQ meshes that represent the surface shapes of freeform architectural roofs [29–31]. Given the excellent performance of deformation based on the stretching energy, we also aspire to make contributions to this field in the future.

## Supporting information

S1 File Code. (PDF)

S1 Fig (TIF)
