# Supplementary material for: NRO-stylization: A novel algorithm for regular octahedral stylization aesthetic modeling of three-dimensional surface mesh
Source: PLoS One. 2024 Oct 29;19(10):e0310242. doi: 10.1371/journal.pone.0310242 (PMC11521310; doi:10.1371/journal.pone.0310242)
Supplement: S1 File — (PDF) [file pone.0310242.s001.pdf]

NRO-stylization

1.Compute Target Normals

```
public List<Vector3D> ComputeNearNormal(TriMesh mesh)
  {
         List<Vector3D> RTN = null;
         if (ConfigDiff.Instance.Cube)
         {
                RTN = ComputeRTNormal();

         }
         else
         {
                RTN = ComputeMeshNormal(GlobalData.Instance.AllMeshes[0]);
         }
         List<Vector3D> result = new List<Vector3D>();

         for (int i = 0; i < mesh.Faces.Count; i++)
         {
                double min = double.MaxValue;
                Vector3D minNormal = new Vector3D(Math.Sqrt(3) / 3f, Math.Sqrt(3) /
3f, Math.Sqrt(3) / 3f);

                Vector3D[] faceNormal = TriMeshUtil.ComputeNormalFace(mesh);
                for (int j = 0; j < RTN.Count; j++)
                {

                       double diff = Math.Acos(faceNormal[i].Dot(RTN[j]));
                       if (diff < min)
                       {
                              minNormal = RTN[j];
                              min = diff;
                              mesh.Faces[i].Traits.SelectedFlag = (byte)(j + 1);
                              // Console.WriteLine(diff);
                       }

                }
                //Console.WriteLine(minNormal.ToString());
                //Console.WriteLine(mesh.Faces[i].Traits.SelectedFlag);
                result.Add(minNormal);
         }

         return result;
```

```
}

private static List<Vector3D> ComputeRTNormal()
{
        List<Vector3D> RTN = new List<Vector3D>();
        RTN.Add(new Vector3D(Math.Sqrt(3)/3f, Math.Sqrt(3)/3f, Math.Sqrt(3)/3f));
        RTN.Add(new Vector3D(Math.Sqrt(3) / 3f, Math.Sqrt(3) / 3f, -Math.Sqrt(3) /
3f));
        RTN.Add(new Vector3D(-Math.Sqrt(3) / 3f, Math.Sqrt(3) / 3f, -Math.Sqrt(3) /
3f));
        RTN.Add(new Vector3D(-Math.Sqrt(3) / 3f, Math.Sqrt(3) / 3f, Math.Sqrt(3) /
3f));
        RTN.Add(new Vector3D(-Math.Sqrt(3) / 3f, -Math.Sqrt(3) / 3f, -Math.Sqrt(3) /
3f));
        RTN.Add(new Vector3D(-Math.Sqrt(3) / 3f, -Math.Sqrt(3) / 3f, Math.Sqrt(3) /
3f));
        RTN.Add(new Vector3D(Math.Sqrt(3) / 3f, -Math.Sqrt(3) / 3f, Math.Sqrt(3) /
3f));
        RTN.Add(new Vector3D(Math.Sqrt(3) / 3f, -Math.Sqrt(3) / 3f, -Math.Sqrt(3) /
3f));
        return RTN;
}
```

2.Compute Rotation Matrix

```
public Matrix3D ComputeRotationRodrigues(Vector3D first, Vector3D second, int
step)
{

        Vector3D cross = first.Cross(second);

        double sin = cross.Length();
        double cos = first.Dot(second);

        //if (cos > 0.99)
        //{
        //        return Matrix3D.IdentityMatrix();
        //}


        double angle = Math.Acos(cos);

        cos = Math.Cos(angle / step);
```

```
sin = Math.Sin(angle / step);

cross = cross.Normalize();
double x = cross.x;
double y = cross.y;
double z = cross.z;

Matrix3D result = Matrix3D.IdentityMatrix();



//result[0, 0] = cos+(1-cos)*x*x;
//result[0, 1] = x * y*(1 - cos) - z * sin;
//result[0, 2] = y* sin+x*z*(1-cos);
//result[1, 0] = z * sin + x * y * (1 - cos);
//result[1, 1] = cos + y * y * (1 - cos);
//result[1, 2] = -x * sin + y * z * (1 - cos);
//result[2, 0] = -y * sin + x * z * (1 - cos);
//result[2, 1] = x * sin + y * z * (1 - cos);
//result[2, 2] =cos + z* z * (1 - cos);




//result[0, 0] = cos*(y*y+z*z) +      x * x;
//result[0, 1] = x * y * (1 - cos) - z * sin;
//result[0, 2] = y * sin + x * z * (1 - cos);
//result[1, 0] = z * sin + x * y * (1 - cos);
//result[1, 1] = cos*(x*x+z*z) + y * y ;
//result[1, 2] = -x * sin + y * z * (1 - cos);
//result[2, 0] = -y * sin + x * z * (1 - cos);
//result[2, 1] = x * sin + y * z * (1 - cos);
//result[2, 2] = cos*(x*x+y*y) + z * z    ;

result[0, 0] = cos * (1 - x * x) + x * x;
result[0, 1] = x * y * (1 - cos) - z * sin;
result[0, 2] = y * sin + x * z * (1 - cos);
result[1, 0] = z * sin + x * y * (1 - cos);
result[1, 1] = cos * (1 - y * y) + y * y;
result[1, 2] = -x * sin + y * z * (1 - cos);
result[2, 0] = -y * sin + x * z * (1 - cos);
result[2, 1] = x * sin + y * z * (1 - cos);
result[2, 2] = cos * (1 - z * z) + z * z;

// result = result.Transpose();
return result;
```

```
}

public virtual Matrix3D[] ComputeRotation(TriMesh mesh)
{
        Matrix3D[] rot = new Matrix3D[mesh.Faces.Count];

        List<Vector3D> RTN = ComputeNearNormal(mesh);

        Vector3D[] faceNormal = TriMeshUtil.ComputeNormalFace(mesh);
        for (int i = 0; i < mesh.Faces.Count; i++)
        {
                rot[i] = ComputeRotationRodrigues(faceNormal[i], RTN[i],
ConfigDiff.Instance.StepPolyCube);

        }

        return rot;

}
```

3. Reconstruct Surface

DeformUtil.Instance.UpdateRightBPoisson(ref curTrans, ref mesh, ref info.RightB3V)

```
            public void UpdateRightBPoisson(ref Matrix3D[] rot, ref TriMesh
BackUpMesh, ref double[][] rightB)
              {
                      double[] weightHF =
LaplaceBuilder.Instance.ComputeCotHalfEdgeDivide2(BackUpMesh).ToArray();
                      foreach (var v in BackUpMesh.Vertices)
                      {

                              Vector3D result = Vector3D.Zero;

                              foreach (var hf in v.HalfEdges)
                              {

                                      Vector3D eij = (hf.FromVertex.Traits.Position -
hf.ToVertex.Traits.Position) ;
```

```
                    if(hf.Face!=null)
                    {
                        Matrix3D rij = rot[hf.Face.Index];
                        result = result + rij * eij * weightHF[hf.Index];

                        //Console.WriteLine("vertex {0}==HF {1}", v.Index, rij
* eij * weightHF[hf.Index]);

                    }
                     if(hf.Opposite.Face!=null)
                    {
                        Matrix3D rji = rot[hf.Opposite.Face.Index];
                        result = result + rji * eij *
weightHF[hf.Opposite.Index];

                        //Console.WriteLine("vertex {0}==HFOpposite {1}",
v.Index, rji * eij * weightHF[hf.Opposite.Index]);
                    }
                }

                rightB[0][v.Index] = result[0];
                rightB[1][v.Index] = result[1];
                rightB[2][v.Index] = result[2];

            }
        }
```

xyz = solver.SolveByPreCompute(info)

```
        public double[][] SolveByPreCompute(InfoSquareConstraints3V info)
        {
            double[][] xyz = null;

            if (Soft == EnumSolverHardSoft.Hard)
            {
                xyz = SolveHardByPreCompute(info);
            }
            if (Soft == EnumSolverHardSoft.Soft)
            {
                xyz = SolveSoftByPreCompute(info);
            }
            if (Soft == EnumSolverHardSoft.HardV2)
            {
```

```
            xyz = SolveByPreComputeQuadratic(info);
        }

        return xyz;
    }
```

DeformUtil.Instance.UpdateMeshPos(mesh, xyz)

```
    public void UpdateMeshPos(TriMesh mesh, double[][] xyz)
    {
        for (int i = 0; i < mesh.Vertices.Count; i++)
        {
            mesh.Vertices[i].Traits.Position.x = xyz[0][i];
            mesh.Vertices[i].Traits.Position.y = xyz[1][i];
            mesh.Vertices[i].Traits.Position.z = xyz[2][i];
        }

        // FixHandlePos();

        TriMeshUtil.SetUpNormalVertex(mesh);
    }
```
