# Supplementary figures and images for: NRO-stylization: A novel algorithm for regular octahedral stylization aesthetic modeling of three-dimensional surface mesh
Source: PLoS One. 2024 Oct 29;19(10):e0310242. doi: 10.1371/journal.pone.0310242 (PMC11521310; doi:10.1371/journal.pone.0310242)

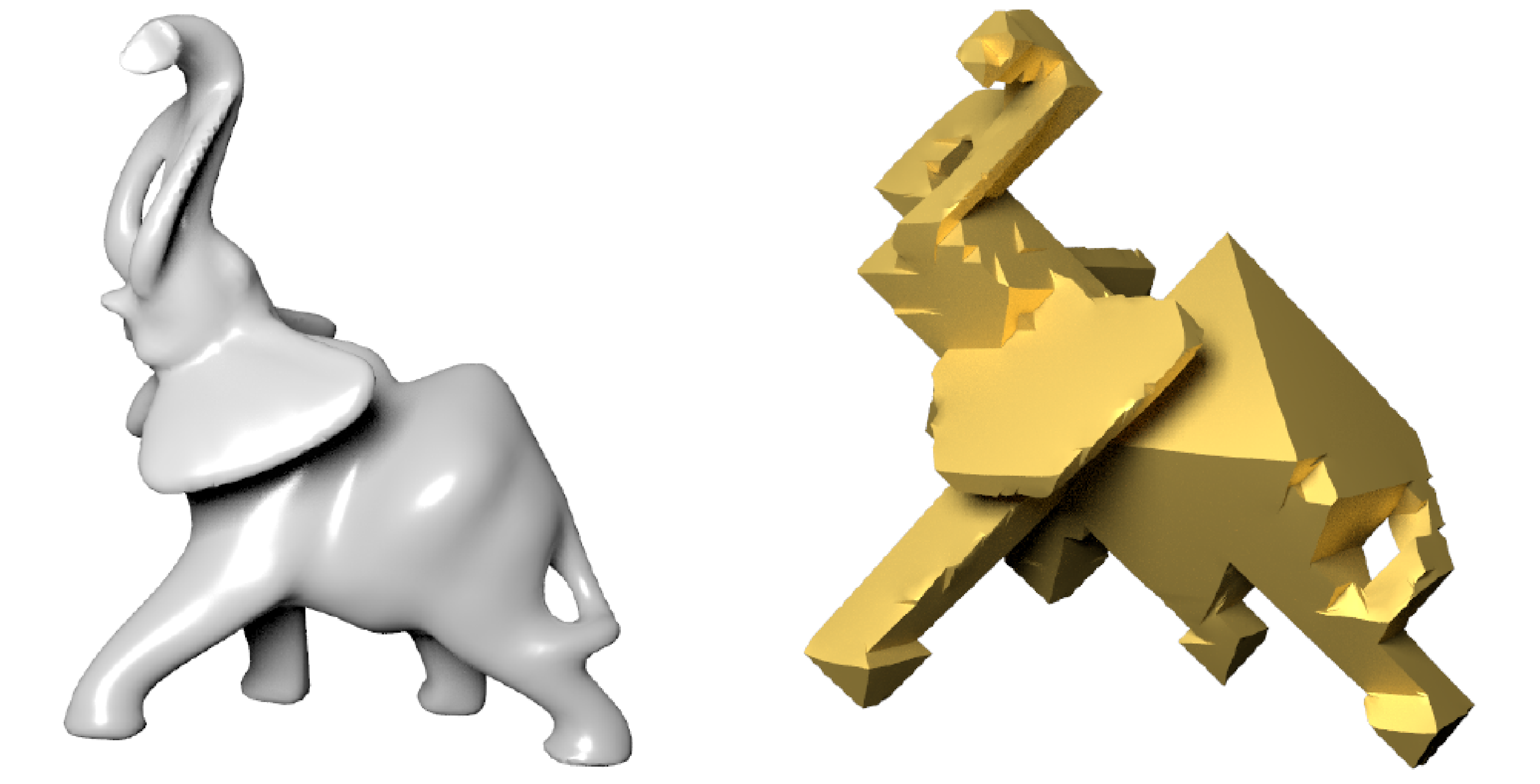

Supplement: S1 Fig — (TIF) [file pone.0310242.s002.tif]
